# Recent advances in understanding lung function development

**DOI:** 10.12688/f1000research.11185.1

**Published:** 2017-05-19

**Authors:** Erik Melén, Stefano Guerra

**Affiliations:** 1 Institute of Environmental Medicine, Karolinska Institutet, Stockholm, Sweden; 2 Sachs’ Children’s Hospital, Södersjukhuset, Stockholm, Sweden; 3 Centre for Occupational and Environmental Medicine, Stockholm County Council, Stockholm, Sweden; 4 Asthma and Airway Disease Research Center, University of Arizona, Tucson, AZ, USA; 5 ISGlobal Center for Research in Environmental Epidemiology, Barcelona, Spain

**Keywords:** Asthma, children, COPD, FEV1, genetics, lung function, tobacco smoke, trajectories

## Abstract

Recent years have witnessed critical contributions to our understanding of the determinants and long-term implications of lung function development. In this article, we review studies that have contributed to advances in understanding lung function development and its critical importance for lung health into adult life. In particular, we have focused on early life determinants that include genetic factors, perinatal events, environmental exposures, lifestyle, infancy lower respiratory tract infections, and persistent asthma phenotypes. Longitudinal studies have conclusively demonstrated that lung function deficits that are established by school age may track into adult life and increase the risk of adult lung obstructive diseases, such as chronic obstructive pulmonary disease. Furthermore, these contributions have provided initial evidence in support of a direct influence by early life events on an accelerated decline of lung function and an increased susceptibility to its environmental determinants well into adult life. As such, we argue that future health-care programs based on precision medicine approaches that integrate deep phenotyping with tailored medication and advice to patients should also foster optimal lung function growth to be fully effective.

## Introduction

Lung development starts *in utero* and may continue throughout childhood ^1, 2^. Evidence is now emerging that several chronic adult diseases—including chronic obstructive pulmonary disease (COPD), which is estimated by the World Health Organization (WHO) to become the third leading cause of death worldwide by 2030 ^3^—may have part of their origins early in life ^4– 9^. These observations fit well with the Developmental Origins of Health and Disease (DOHaD) concept that describes how early life exposures may have a long-term impact on diseases in adulthood ^5, 6, 10– 12^. Within this framework, we review studies that have contributed to recent advances in understanding lung function development and its critical importance for lung health into adult life. The review covers key determinants, from genetics to early life events and environmental exposures, and focuses primarily on lung function trajectories from childhood to adulthood.

## Factors affecting lung function growth

### Genetic factors influencing lung function

The genetic determinants for lung function have been evaluated in numerous candidate gene studies over the years and in the last 5 to 10 years successfully in large genome-wide association studies (GWASs). In the most recent GWAS on almost 50,000 subjects from the UK Biobank followed by replication in 95,000, it was concluded that the number of independent genetic associations with any lung function parameter—forced expiratory volume in one second (FEV _1_), forced vital capacity (FVC), or the FEV _1_/FVC ratio—is now 97, representing loci across the whole genome ^13^. The total heritability explained by these 97 signals was estimated to 9.6% for FEV _1_, 6.4% for FVC, and 5.2% for FEV _1_/FVC. Importantly, most of the identified single-nucleotide polymorphisms (SNPs) seem to influence lung function in both children and adults, a pattern that has been observed in several studies ^14– 18^. Many of the identified SNPs have also been associated with COPD in previous studies ^19^. Attempts have been made to identify gene variants predisposing to different lung function trajectories from childhood to adulthood, and in the US CAMP study a SNP on chromosome 8 (rs4445257) between *CSMD3* and *TRPS1* was found to be significantly associated with a normal-growth, early-decline pattern ^20^. However, in adults, genetic variants known to be strongly associated with cross-sectional lung function show little or no association with the rate of lung function decline over time ^21^.

Recent understanding that the genetic determinants of lung function operate across the life cycle lends support to the hypothesis that lung function trajectories from childhood to adulthood are at least partly defined at birth and early life. Intriguingly, pathway analyses show enrichment in lung function genes for developmental processes, and functional genetic and proteomic analyses of fetal lung samples also show that several of these genes (for example, *TMEM163*, *FAM13A*, *HHIP*, *CDC123*, *PTCH1*, and *RAGE*) affect lung development already at the embryonic stage ^22, 23^. As such, it is possible that variants associated with lung function and respiratory disease in adulthood may actually influence risk through mechanisms that are at least partly related to lung development.

### The relation of preterm birth to lung function

Undisputable examples of early life effects are chronic lung disease (for example, bronchopulmonary dysplasia) and lung function impairment in individuals born very prematurely (fewer than 32 gestational weeks) ^24– 27^. Recent studies also show that late to moderate preterm birth (32 to 36 gestational weeks) is associated with significant lung function deficits at least up to adolescence, particularly for airflow limitation indices measured as FEV _1_ and FEV _1_/FVC ^11, 12^. Lung function catch-up (that is, recovery of deficits observed during childhood) has been reported in some ^28, 29^ but not all ^11^ studies. The long-term clinical relevance of small to moderate lung function deficits in childhood related to preterm birth and perinatal events is not known. However, concern has been raised as to whether individuals born preterm, especially those born extremely preterm, may be at risk of developing COPD-like phenotypes later in life ^30^. In addition, it is still unclear whether preterm birth is associated with a more rapid age-related decline in lung function in adulthood. **

### Relevance of environmental exposures

Air pollutants may induce airway inflammation, increased airway responsiveness, and lung damage and this is partly due to generation of free radicals and oxidative stress. Exposure to traffic-related air pollution has been negatively associated with lung growth and lung function (primarily FEV _1_) in children and young adults in several studies, leading to increased risk of clinically important deficits ^31– 34^. In studies from the Swedish BAMSE (Barn/children Allergy Milieu Stockholm Epidemiology) cohort, conducted in the Stockholm area with air pollution exposure levels well below the current WHO guidelines, exposure during the first year of life seemed to have the largest impact on later lung function ^31, 32, 35^. Early life exposure is also associated with increased risk of asthma throughout childhood ^36^, and interaction with genetic factors related to COPD has recently been reported ^37^. These studies indicate a vulnerable time window early in life, and this is consistent with the DOHaD hypothesis. However, it is still unclear whether early life exposure has long-term effects into adulthood. Reports from the Children’s Health Study in California show convincing data on the negative impact on lung function indices from air pollution exposures later during childhood and adolescence ^38, 39^. Notably, improvements in air quality have been shown to have positive effects on lung function growth between 11 and 15 years, indicating that later exposures are very likely to be of importance ^40^.

One of the most well-studied risk factors for respiratory disease is tobacco smoke exposure. Several studies have reported maternal smoking during pregnancy as a major risk factor for impaired lung development ^41– 43^. The epidemiological associations have been supported by experimental studies showing structural lung defects, hyperplasia of neuro-endocrine cells, and decreased lung growth in offspring exposed to tobacco smoke *in utero* ^44, 45^ as well as human data reporting consistently altered epigenetic profiles in children of mothers who smoked during pregnancy ^46^. Secondhand tobacco exposure later during childhood has also been associated with persistence of respiratory symptoms into adult life ^47, 48^. Indeed, studies reporting interactive effects between parental and active smoking in affecting FEV _1_ decline and COPD risk are among the best illustrative examples of how risk factors from early and adult life may have detrimental joint health effects. In cross-sectional studies of active smokers, having a mother who also smoked was linked to airflow limitation ^49^ and early-onset ^50^ and severe ^51^ COPD and these observations have been recently expanded to decline of lung function in longitudinal studies, as described in the following sections. Despite these well-known negative health effects and anti-smoking campaigns worldwide, smoking during pregnancy and elsewhere remains a major public health challenge.

### Dietary factors and physical activity

Our constantly changing lifestyle has a broad impact on health and well-being. Diet and physical activity are modifiable factors that may influence lung development and respiratory disease across the life span. Systematic reviews show evidence of a beneficial effect of fresh fruits, and antioxidant vitamins on recurrent wheeze and asthma, but most evidence stems from cross-sectional studies and there is a need for more well-designed randomized controlled trials (RCTs) ^52^. In particular, vitamin D has been implicated to have a key role in lung development ^53^, and maternal deficiency has been reported to be associated with impaired lung development in school-aged offspring ^54, 55^. Although recent RCTs have shown that fish oil-derived fatty acid, vitamin C, or vitamin D supplementation during pregnancy may have beneficial effects on offspring respiratory health ^56– 59^, there are currently no consensus guidelines or recommendations of specific diets or supplements for lung function improvement in children. Results from ongoing clinical studies in this area are much anticipated.

Physical activity and fitness have been associated with childhood lung function in some ^60, 61^, but not all ^62^ studies. However, recent longitudinal data show that achieving increased fitness from young adulthood to middle age is associated with less decline in lung health over time ^63^. These results are encouraging for patients with a lung disease.

### Role of lower respiratory tract infections

From a clinical point of view, it is well known that infants with severe bronchiolitis triggered by, for example, respiratory syncytial virus (RSV) or rhinovirus (RV) are at risk of later asthma or lung function impairment, or both. Longitudinal cohorts show that children with virus-induced wheezing symptoms during the first years of life may outgrow their symptoms later in childhood but as a group do not completely overcome their lung function impairment ^64– 70^. If a child develops recurrent wheeze or asthma following lower respiratory tract infection (LRTI) in infancy, he or she may be at increased risk for a further deterioration of lung function (the two-hit hypothesis ^65, 71, 72^) or develop an increased susceptibility to later noxious environmental exposures as discussed below. Whether these early and later lung function insults are related to independent risk factors and pathways or instead share a genetically determined susceptibility remains to be elucidated.

The underlying mechanism for the association between respiratory tract infection in early life and later respiratory morbidity is not clear. Proposed mechanisms include modulation of the immune response, direct airway damage, pre-existing deficits in lung function, and genetic susceptibility—highlighting different aspects of cause and effect ^73– 77^. Some studies suggest that specifically an early RV wheezing attack or bronchiolitis event is a marker of risk for later asthma ^78^, whereas others suggest that the number, not the particular viral species, of LRTI episodes in the first years of life is of primary importance for later asthma development ^79^. As a possible mechanism linking RV to asthma, the gene encoding for the RV receptor (C) is the asthma susceptibility gene *CDHR3* on chromosome 7q22. *CDHR3* was first identified in a GWAS on severe asthma in children ^80^, and only later did experimental work lead to the identification of CDHR3 as the virus receptor ^81^. In addition, variants at the 17q21 asthma locus ( *ORMDL3*) have been specifically associated with asthma in children who had had RV wheezing illnesses early in life, connecting genetic susceptibility, RV infection, and asthma development ^82^.

## Lung function trajectories from childhood into adult life

### Long-term effects of lung function development into adult life

A large growing body of evidence indicates that lung function development *in utero*, infancy, and childhood may have long-lasting effects on respiratory health throughout the life span. Most studies have addressed trajectories of lung function from childhood into adult life by focusing on FEV _1_ and the FEV _1_/FVC ratio as indices of airflow limitation. Consequently, airway obstruction—which is defined by an abnormally low FEV _1_/FVC ratio and represents the hallmark of COPD ^4^—has been by far the most extensively studied spirometric pattern and will be the main focus of this section, although we argue that studies addressing the early origins of spirometric restriction ^83– 86^ are also needed because of its remarkable prevalence, morbidity, and mortality burden.

**Figure 1.**
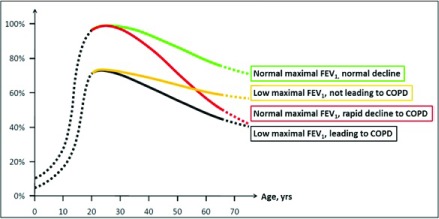
Lung function trajectories to chronic obstructive pulmonary disease (COPD). The figure represents four lung function trajectories identified in the study by Lange *et al*. ^7^ based on levels of forced expiratory volume in one second (FEV _1_) before the age of 40 years (below or above 80% of predicted value) and the presence or absence of Global initiative for chronic Obstructive Lung Disease (GOLD) grade of at least 2 COPD at the end of follow-up. The y-axis represents the percentage of expected maximally attained FEV _1_. Modified from 7.

The possible contribution of childhood factors on the natural history of obstructive lung diseases across the life span has been the topic of much debate for decades ^87– 90^. In recent years, the importance of a full growth to maximal lung function in childhood has been reinforced by conclusive evidence that COPD can develop in mid to late adult life through at least two main trajectories: by the classic trajectory of an accelerated FEV _1_ decline in adulthood following a normal lung function development in childhood (“Rapid FEV _1_ decline” trajectory, red line in Figure 1, modified from reference 7) or alternatively by a low maximal lung function attained by the beginning of adult life without necessarily an accelerated FEV _1_ decline thereafter (“Low maximal FEV _1_” trajectory, black line in Figure 1). Strikingly, in a recent study ^7^ that included three large prospective cohorts, about 50% of participants with COPD exhibited features compatible with the latter trajectory, suggesting that lung function development in childhood may play a substantially more relevant role in COPD susceptibility than traditionally thought.

### Early origins of the low maximal lung function trajectory

Among the characteristics associated with a persistently low lung function trajectory are the presence of recurrent wheezing, asthma, and asthma-related phenotypes in childhood. As several longitudinal birth cohorts have now entered their adult years, their findings have been consistent in showing substantial tracking of asthma-related lung function deficits from childhood into adulthood but no evidence of accelerated decline thereafter ^91, 92^. Notably, studies that have measured indices of airway resistance and airflow at earlier ages ^71, 72^ have demonstrated that, although deficits of lung function can be detected as early as one month after birth, most children who have persistent wheezing and asthma by early school age experience a progressive deterioration of their lung function deficits as they transition from infancy to school age. These findings suggest that *in utero* life, infancy, and early childhood may be critical windows of opportunity for early prevention of long-term sequelae of childhood asthma on lung health.

In this context, several recent studies have provided strong evidence that the lung function deficits associated with severe childhood asthma may lead in a subgroup of patients to the development of COPD. In the Melbourne study ^93^ and Scottish WHEASE (What Happens Eventually to Asthmatic children: Sociologically and Epidemiologically) cohort ^94^, children with asthma—particularly if severe—had a striking increase in the risk of having a post-bronchodilator FEV _1_/FVC ratio of less than 70% by the age of 50 to 65 years. Consistent with these epidemiological data, up to 11% of children with persistent mild to moderate asthma in the clinical CAMP study were found to develop COPD according to spirometric criteria by age 30 years ^8^. In that study ^8^, participants were classified into four groups on the basis of visual inspection of their lung function trajectories from childhood into young adult life: normal growth, normal growth and early decline, reduced growth, and reduced growth plus early decline. In line with findings from the studies described above, nearly 85% of the cases who went on to develop COPD by young adulthood occurred among participants who experienced a reduced lung function growth in childhood and adolescence. Interestingly, despite the relatively short follow-up in adulthood, an early start of lung function decline appeared to also carry an increase in COPD risk. Future studies should address to what extent a short plateau phase or early decline of lung function may also contribute to COPD development among patients with asthma.

It should be noted that the tracking of lung function from childhood into adult life is not observed only among individuals with asthma but has been repeatedly shown in samples from the general population. Tasmanian children who were in the lowest quartile of FEV _1_/FVC at age 7 years had a six- to 16-fold increase in their odds for COPD in the absence or presence of concomitant asthma by age 45 ^9^. In the population-based Tucson Children’s Respiratory Study, participants in the lowest quartile of airway function in early infancy had significantly lower values for FEV _1_ and FEV _1_/FVC up to age 22 as compared with participants in the upper three quartiles ^95^, and a distinct group of individuals with persistently low lung function between the ages of 11 and 32 years (a large proportion of whom did not have asthma) could be identified by using latent class analysis ^10^. Interestingly, participants in this impaired lung function trajectory, as compared with participants in the normal lung function trajectory, were nearly twice as likely to have had RSV lower respiratory tract infections in the first three years of life. Thus, we argue that multiple host factors, exposures, and events that have a direct impact on lung function at any developmental stage may in principle contribute to put a child in a trajectory of persistently low lung function into adult life, with a very broad range of effect magnitude. What makes some children robust to the effects of these risk factors and why some children may develop only initial lung function deficits in response to these exposures that are transient and eventually overcome them as they enter adult life remain largely unknown and, as discussed in the concluding section, a question with critical implications for prevention.

### Early origins of rapid lung function decline

Evidence is beginning to emerge that early life factors may also predispose to an accelerated decline of lung function in adult life (red line in Figure 1). Among adult participants in the European Community Respiratory Health Survey, recalling the presence of “disadvantage” factors in childhood (that is, maternal asthma, paternal asthma, childhood asthma, maternal smoking, or childhood respiratory infections or a combination of these) was associated not only with the presence of airflow limitation but also with an accelerated decline of FEV _1_^96^. Similarly, in the adult CARDIA (Coronary Artery Risk Development in Young Adults) Study, a low childhood socioeconomic status (as assessed by self-reported parental education) was associated with a steeper decline of both FEV _1_ and FVC in adult life, although the specific factor(s) involved in explaining this association could not be identified ^97^.

Despite this evidence, data in support of direct effects of early life factors on accelerated decline of lung function in adult life are still sparse and consequently the magnitude and consistency of these associations unclear. This may be due to the inherent methodological difficulties of assessing these effects within a longitudinal study design or to the possibility that these effects are synergistic with other exposures in adult life. Although both scenarios are likely to be correct, the latter is supported by the recent and growing body of evidence from epidemiological and experimental studies suggesting that early life factors may increase susceptibility to the effects of adult life exposures in affecting lung disease. The presence of RSV LRTIs in the first 3 years of life and other childhood factors have been shown to interact with active smoking and occupational hazards in adulthood to affect respiratory symptoms and asthma risk ^98, 99^. Most interestingly, early exposure to maternal and parental smoking has been shown to enhance susceptibility to smoking-related accelerated decline of lung function in adulthood ^100, 101^. In the Tucson cohort, participants were classified on the basis of exposure to parental smoking (assessed at birth) and active smoking in adult life ^101^. Between the ages of 11 and 26 years, participants with exposure to both parental and active smoking had the steepest decline in sex-, age-, and height-adjusted residuals of FEV _1_/FVC, FEV _1_, forced expiratory flow at 25% to 75% of FVC (FEF _25–75_), and FEF _25–75_/FVC. In contrast, no significant deficits were seen at this young age among participants who were exposed to only parental or only active smoking, indicating that early life (or *in utero*) exposure to environmental tobacco smoke increases susceptibility to the deleterious effects that active adult smoking will have on lung health. The exact mechanisms through which exposures to tobacco smoke in early and adult life interact with each other in affecting susceptibility remain largely unknown, as does the extent to which these synergist effects with adult life exposures may apply to other early life factors. Epidemiological and clinical studies aimed to dissect systematically interactive effects between early and adult life events on lung health outcomes are warranted.

## Conclusions

Recent years have witnessed critical contributions to our understanding of the determinants and long-term implications of lung function development. In addition to further delineating the role of genetics, perinatal events, childhood environmental exposures, lifestyle, infancy LRTIs, and persistent asthma phenotypes, these contributions have conclusively demonstrated that lung function deficits that are established by school age may track into adult life and increase the risk of adult lung obstructive diseases, including COPD. Furthermore, these contributions have provided initial evidence in support of a direct influence by early life events on an accelerated decline of lung function and an increased susceptibility to its environmental determinants (for example, tobacco smoke) well into adult life. Thus, this evidence indicates early life as a critical time that may contribute to set the pace of lung aging processes that will take place several decades later.

Although this conclusion highlights the critical importance of developmental age, it should not undermine in any way the importance of behavioral and environmental risk factors for obstructive lung diseases that take place in adult life. Indeed, avoidance of such risk factors in both childhood and adulthood needs to play a critical role in primary to tertiary prevention. We argue that tailoring risk profiles and intervention strategies based on information from both early and adult life factors may provide a significant improvement in the way we prevent and treat lung disease.

**Figure 2.**
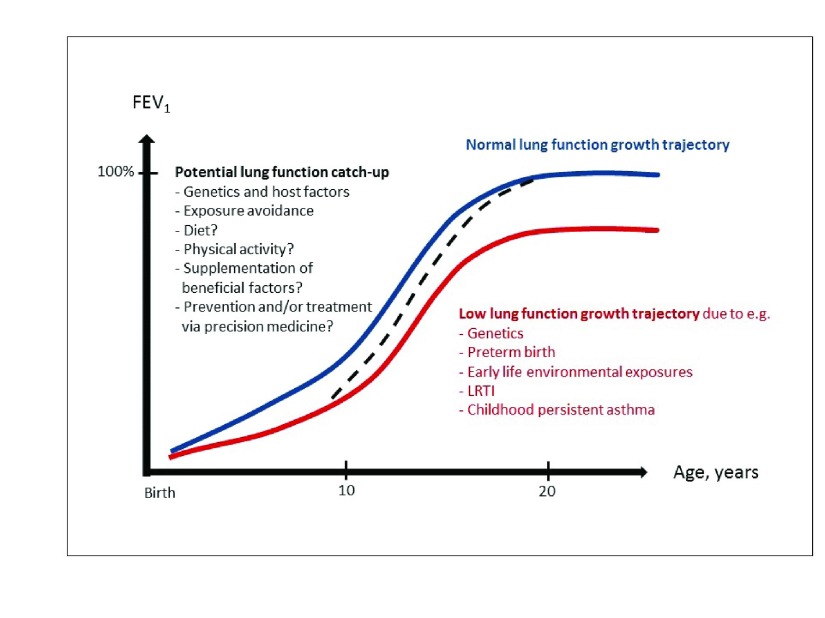
Lung function trajectories from childhood to adult life. The blue line represents a normal lung function growth, the red line represents a low lung function trajectory and associated risk factors, and the black dotted line represents lung function catch-up from childhood to adulthood. Potential beneficial factors for lung function catch-up are listed in black text. FEV _1_, forced expiratory volume in one second; LRTI, lower respiratory tract infection.

As a concluding note, we point out that although great effort has been directed to characterize trajectories of lung function deficits and to identify risk factors associated with lung function impairment, less is known about factors influencing optimal lung function growth or recovery from early deficits or after early insults such as preterm birth or a severe LRTI ( Figure 2). Indeed, whether (and to what extent) lung function catch-up occurs in groups of children with early deficits has been insufficiently studied to date. Identifying effective strategies—apart from avoiding obvious risk factors like tobacco smoke, air pollution exposure, and recurrent respiratory infections—to enhance this early catch-up would have critical implications. The roles of dietary components and supplementations ^52, 53^ and those of physical activity and fitness ^60– 62^ are among those being investigated. Molecules that may play a direct protective role in the lung—such as the club cell secretory protein (CC16)—are also being evaluated in epidemiological ^102, 103^ and clinical ^104, 105^ studies and may hold promise as future therapeutic strategies.

In this context, the major advances from recent years in understanding the genetic and molecular components of lung function not only will pave the way for new asthma and COPD drugs but also provide critical knowledge to help doctors and caregivers to optimize their patients’ lung health. Future health-care programs based on precision medicine approaches that integrate deep phenotyping with tailored medication and advice to patients should also foster optimal lung function growth to be fully effective.
